# Development and Educational Effectiveness of a Mixed Reality (MR) Program to Support Clinical Judgment in the Observation of Postoperative Patients

**DOI:** 10.3390/healthcare12232357

**Published:** 2024-11-25

**Authors:** Naoya In, Rei Wakamatsu, Haruma Miyakawa, Chie Kushima, Xiaoshuai Chen, Toshiko Tomisawa

**Affiliations:** 1 Graduate School of Health Sciences, Hirosaki University, 66-1 Hon-cho, Hirosaki 036-8564, Japan; c.kushima@hirosaki-u.ac.jp (C.K.); tmtott@hirosaki-u.ac.jp (T.T.); 2 Faculty of Science and Technology, Hirosaki University, 3 Bunkyo-cho, Hirosaki 036-8561, Japan; h20s5077@hirosaki-u.ac.jp; 3 Graduate School of Science and Technology, Hirosaki University, 3 Bunkyo-cho, Hirosaki 036-8561, Japan; h23ms544@hirosaki-u.ac.jp (H.M.); chen@hirosaki-u.ac.jp (X.C.)

**Keywords:** nursing education, clinical judgment, mixed reality (MR), simulation, postoperative patient care, observation

## Abstract

Objective: The aim of this study was to develop and evaluate the effectiveness of a clinical judgment support program using mixed reality (MR) for the observation of postoperative patients. Methods: This study employed a randomized controlled trial design, with 34 fourth-year nursing students as participants. The students were randomly allocated into two groups: a traditional simulation group (Sim group, *n* = 17) and an MR group (*n* = 17). Both groups were tasked with observing postoperative patients and making clinical judgments. The Sim group engaged in patient observation through classical simulation, followed by a debriefing session with the investigator. The MR group observed the patient according to the procedure displayed on HoloLens2 and conducted the self-debriefing using the developed program. Key outcome measures included educational time, the number of items observed, motivation for learning, satisfaction, confidence, and participant feedback. Results: The results indicated that the MR group was able to observe a more significant number of observation items. Additionally, while the simulation time was longer in the MR group, the debriefing time was shorter compared to the Sim group. Psychological safety was higher in the MR group, whereas the Sim group, which had individualized debriefing opportunities, reported significantly increased confidence and reduced anxiety. Conclusions: The findings suggest that utilizing MR-based materials for teaching postoperative patient observation is more efficient and effective in educating novice nursing students.

## 1. Introduction

Postoperative patients often have unstable conditions due to the impact of surgery, requiring nurses to have strong clinical judgment skills to anticipate complications and provide care. Clinical judgment involves making appropriate decisions based on the patient’s needs and concerns and adjusting approaches as necessary. This is a vital skill for nurses [1].

In the observation of postoperative patients, clinical judgment must be exercised based on knowledge and skills related to diseases, treatments, and complications and refined through trial and error. Previous research has highlighted the care algorithms experienced nurses employ [2]. To effectively develop clinical judgment, specialized education, particularly experiential learning, is essential [1]. Simulation-based training that allows learners to experience expert clinical decision-making, along with observation and mentorship during clinical practice, is necessary to acquire these skills [3,4].

However, several challenges have been identified, including the limitations of using simulated patients and simulators, the inability to perform certain highly invasive care procedures, and the need for human resources, skills, materials, and resources to run and supervise these programs [4]. Additionally, the COVID-19 pandemic has restricted in-person clinical training and mentorship, significantly reducing experiential learning opportunities crucial for developing clinical judgment [5]. As a result, students who have completed their programs have expressed concern about their readiness for graduation [6]. Therefore, there is a growing need to develop new experiential learning programs for nursing students that will enable them to effectively cultivate clinical judgment in observing postoperative patients.

VR (virtual reality) is a technology that immerses users in a computer-generated virtual world, allowing them to experience environments similar to the real world [7]. AR (augmented reality) superimposes virtual objects onto the real world, making them appear as if they exist within the physical environment [8]. MR (mixed reality) combines elements of both the real and virtual worlds, enabling users to interact with virtual objects as if they were part of the physical environment, offering an interactive and immersive experience. While VR makes it difficult to represent structures and creates a sense of unreality, AR and MR can create a true immersion by combining virtual reality with real objects, adding a sense of reality by allowing actions to be taken in the real world. Furthermore, MR technology has enhanced learning retention, performance, and engagement among learners [9]. In medical and nursing education, MR is used for skills training, including airway management, surgical procedures, emergency decision-making, patient assessment, and procedural training in medical and nursing education [10,11,12]. Research has shown that it positively impacts clinical outcomes, learner satisfaction, confidence, and motivation. MR also facilitates the integration of basic knowledge and skills with clinical judgment and practice. Additionally, it has the advantage of causing less motion sickness than VR [12]. Previous studies on MR-based education for nursing students found that MR was used to provide practical training to develop communication skills [13], to foster critical thinking and improve students’ clinical reasoning and judgment skills [14], and used in psychiatric nursing education to improve patient comprehension and confidence [15]. However, no studies have used MR to educate clinical judgment in the observation of postoperative patients.

Therefore, the purpose of this study is to examine the effectiveness of the MR program to support clinical judgment in the observation of postoperative patients by comparing it with conventional simulation education. This program will enable students to experience and learn the clinical judgment of skilled nurses through visualization and is expected to improve the practical nursing skills of the learners.

## 2. Materials and Methods

### 2.1. Trial Design

The design of this study was a randomized controlled trial.

### 2.2. Subjects

The subjects in this study were fourth-year nursing students in University X School of Health Sciences. All of the subjects had obtained credits in Foundational Nursing Science, Core Nursing Courses: Overview, Specialized Topics, and Nursing Practicum (adult, pediatric, and maternal nursing), and had basic knowledge of observation of postoperative patients, which was the case problem of this study.

Data were collected in the laboratory of the researcher’s educational institution. The experimental environment was designed to simulate a hospital room where actual postoperative patients were observed and were equipped with a bed, a biological monitor, an oxygen administration set, a suction set, an IV stand, and an infusion pump. The simulation space was separated from monitoring by the researcher through partition.

### 2.3. Intervention

#### 2.3.1. Definition of Terms

The terms in this study were defined as follows:

- Observation: Physical assessment in postoperative patients and collection of subjective and objective data to judge the possible onset of postoperative complications.
- Clinical judgment: Based on a previous study [2], evaluate normal or abnormal subjective and objective data and combine multiple observations to judge patient stability and postoperative complications.

#### 2.3.2. Methods

**Figure 1:**
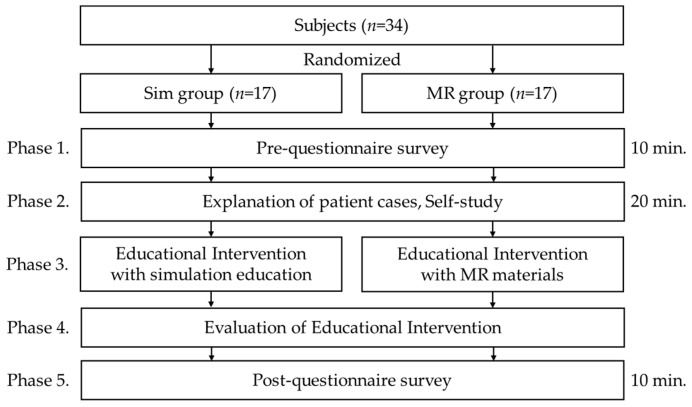
Research flow.

The experiment was conducted following the research flow shown in Figure 1.

Phase 1. Pre-Questionnaire Survey

A self-administered questionnaire was used to assess basic attributes (Age, Gender, Practical experience, Good or poor at observation) and understanding, anxiety, and confidence regarding observation of postoperative patients (5 items).

Phase 2. Explanation of Patient Cases, Self-Study

After explaining the case, the participants were given 20 min for self-study to visualize the procedure and content of observation on the postoperative patient and the case, freely using materials, textbooks, websites, etc., that the subjects would usually study. It was explained to the participants that they would not use communication devices to contact others during self-study. In addition, the researcher monitored the study content to ensure that no MR material content or direct responses to tasks were used.

Phase 3. Educational Intervention

Educational intervention on observation of patients postgastrectomy was conducted for each subject using the simulation material (Sim group) and MR materials (MR group).

- Sim group: The subjects received simulation education from the researcher acting as a facilitator through the teaching materials described below (see Section 2.3.5).
- MR group: The subjects were educated using the MR materials developed. The researcher assisted in fitting the device and explaining how to use it, but MR materials conducted the education itself, and the researcher only conducted the monitoring.

Phase 4. Evaluation of Educational Effectiveness

① Observation Practice

After learning through the simulation and MR teaching materials, the subject was asked to practice observation of a postoperative patient for the same case using the high-functioning simulator as the patient. As in the simulation education, the researcher presented photographs of observation items that could not be reproduced on the high-functioning simulator, and the researcher conducted the patient’s responses. The subjects’ practice was video recorded, and the observation items were monitored.

② Confirmation of Practice

The video of the practice was viewed with the subject, who was asked to review the items observed and number them in the order in which they were observed. They were also asked to select from multiple options that included various levels of judgment criteria for the results of their judgments during the observation.

Phase 5. Post-Questionnaire Survey

A self-administered questionnaire was used to assess understanding, anxiety, and confidence in observing postoperative patients, satisfaction and confidence in learning, motivation to learn, and evaluation of MR materials.

#### 2.3.3. Scenario

The case in which participants do simulation is shown in Table 1. It was built based on the postoperative patient with gastrectomy (Table 1). Based on prior research and taking into account the observation priorities of experienced nurses, 70 observation items and seven postoperative complications judgment items were developed for postgastrectomy patients (Table 2).

#### 2.3.4. Simulator

A high-fidelity simulator (Nursing Anne: Laerdal, Stavanger, Norway) equipped with an oxygen mask, a nasogastric tube (left nostril), a Foley catheter, a drain (right abdominal area), an intravenous line (left forearm), a gauze dressing (abdominal), and elastic stockings were used in this study.

#### 2.3.5. Teaching Materials of Educational Intervention

Educational Intervention was performed through simulation and MR materials.

(1) Simulation

The simulation materials were developed in accordance with the “Best Practice Standard: Simulation” established by the International Nursing Association for Clinical Simulation and Learning [16]. In addition, a nursing faculty member with expertise in simulation education supervised the development process to ensure the validity of the educational content. The contents were as follows:

- Briefing

An explanation of how the simulation was performed was provided. The color tone and condition of the patient’s wound, back, buttocks, heel, and toes, which the simulator could not represent, were observed in photographs presented by the researcher at the time the observation was required. In addition, when the subject speaks to the patient, the researcher responds as the patient. No statements were required, as the content of the observations, results, and judgments were to be confirmed later. The results of vital signs and other measurements were explained to the patients so that they could check the results on the monitor. Although the above differs from the practice in the clinical setting, the researcher explained that the subjects should practice the same way they usually do when working with patients.

- Simulation

The subjects were asked to observe a high-functioning simulator that reproduced the conditions of the case study patients. During the implementation, the researcher monitored the subject’s practice and wrote down the items on a whiteboard each time they were observed. The indicators of the observed items were by the 70 expected observation items.

- Debriefing

Based on the observation items written out on the whiteboard, the participants were asked to speak their thoughts on the procedures and judgments that were implemented. The correct observation procedures and clinical judgments were then explained using the whiteboard.

(2) MR

① Hardware

HoloLens2 (Microsoft, Washington, DC, USA)

② The Procedure of MR Creation

Researchers who had experience in a postoperative clinical setting made the structure of the MR program. Science and technology researchers with programming skills created an MR program using Unity 2022 and Visual Studio 2019 and implemented it in HoleLens2.

③ The Structure of MR Material

The MR program is organized as follows:

- Briefing

**Figure 2:**
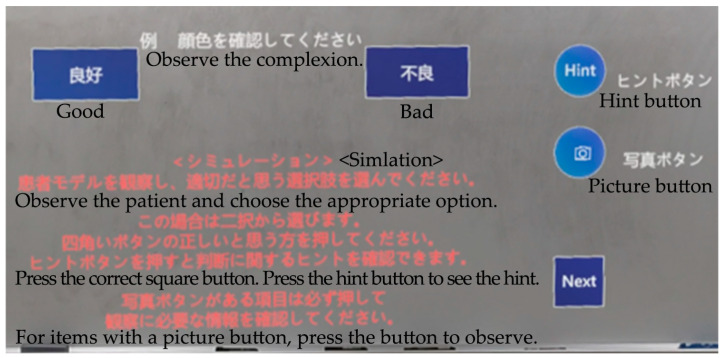
A view of the subject with a briefing on how to use the program, including following the instructions to observe the patient and select the appropriate option, pressing the hint button to see hints about the judgment, and pressing the photo button to observe the patient.

The briefing included instructions on how to conduct the simulation using MR-based educational materials. Participants were instructed to wear the HoloLens2 and follow the displayed observation items, pressing the hint button when necessary to review the observation methods and selecting the appropriate observation results from the options given. It was explained that vital signs should be checked on the monitor. Additionally, participants were informed that verbal communication of their observations, results, and judgments was unnecessary, as these would be reviewed later. The actual program screen and explanatory text were presented on the HoloLens2 screen (Figure 2). Furthermore, before starting, the participants practiced the operation by touching the buttons displayed in the space in front of them.

- Simulation

**Figure 3:**
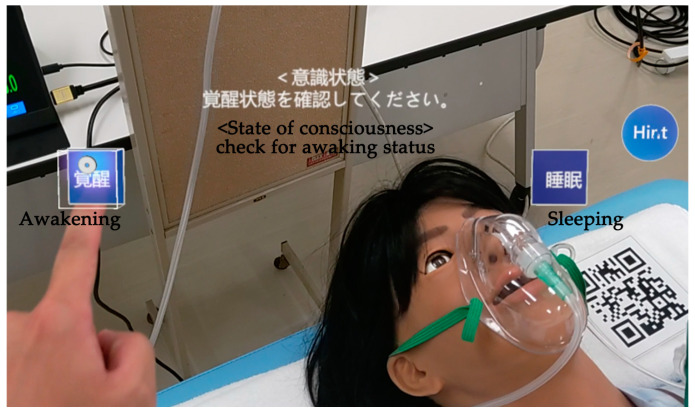
The subject’s view during the observation and decision-making process.

**Figure 4:**
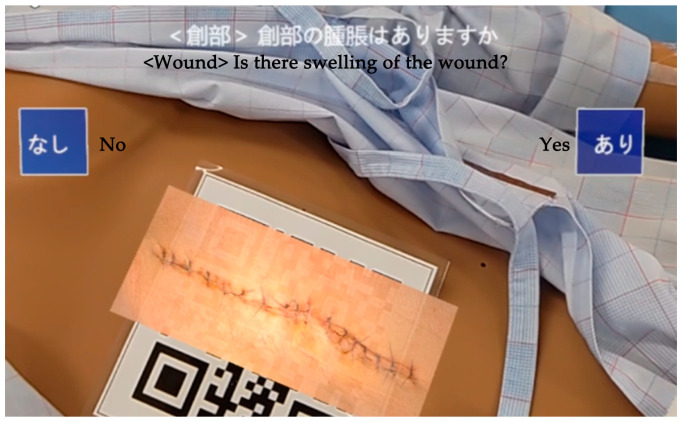
The subject’s view observing a picture of the wound displayed.

According to the 70 observation items and seven postoperative complication decision items created, step-by-step instructions were displayed in augmented reality via HoloLens2, guiding participants to observe postoperative patients accordingly. If participants encountered difficulties understanding the observation or judgment process, a hint button provided visual assistance in the form of text or images. Multiple-choice buttons for each observation were displayed for various levels of judgment criteria (Figure 3). The next observation item was not presented until the current item’s observation and judgment were completed. The program could not be concluded until all observations were fully completed. For aspects such as skin conditions and color tones that the simulator could not replicate, including the patient’s face, surgical wound, heels, and toes, QR codes approximately 10 cm square were placed on the left side of the face, the abdominal wound, and the lower part of each foot. When recognized by HoloLens2, these QR codes triggered the display of actual patient images (Figure 4). Image buttons appeared on HoloLens2 when observation was necessary for the back and buttocks, and participants could view images by selecting the appropriate button.

Subjects were required to observe a high-fidelity simulator that reproduced the patient’s condition using the MR program.

- Debriefing

**Figure 5:**
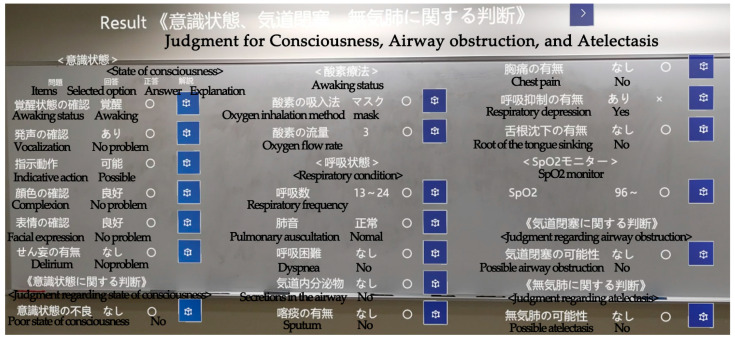
A view of the subject during debriefing, each with a list of observation items, selected options, correct or incorrect answers, and a button to view the explanation.

After the completion of the observation in simulation, the items observed by the subject and the correct or incorrect result of the selection were displayed on four pages (Figure 5). The display screen was designed to allow the subject to select and manipulate the part of the screen that they wanted to see. In addition, an explanation button was provided for each observation and judgment item to display the correct method of observation and judgment, as well as text regarding the results. Subjects were asked to self-learn until they understood the observation procedures, contents, and clinical judgment on their own.

④ Validation

The program was developed by nursing faculty with at least five years of experience observing postoperative patients in intensive care units to check the validity of the content.

### 2.4. Outcomes

1. Education Time

Simulation time, Debriefing time, and Total time were measured to evaluate the educational intervention with simulation and MR materials.

2. Effectiveness of Educational Intervention

The effectiveness of the educational intervention was evaluated by measuring the number of observation and postoperative complication judgment items performed. The number of items was based on a paper list of 70 observation items and seven postoperative complication judgments, and the items performed were checked while watching the video of the observation practice.

3. Understanding, Anxiety, and Confidence Regarding Observation of Postoperative Patients

The subjects were asked to rate their understanding, anxiety, and confidence on a 5-point scale from “−2: Strongly disagree” to “2: Strongly agree” for the following five items originally created; “I can explain observation procedures”, “I can explain observation methods”, “I can explain the judgment required to observe”, “I am not anxious about the observation”, “I am confident in my observations” (Questionnaire).

4. Satisfaction and Self-Confidence in Learning

The Japanese version of the “Student Satisfaction and Self-Confidence in Learning” developed by the National League for Nursing was used with permission from the developer [17,18]. This scale consists of 13 items related to satisfaction with and confidence in simulation education. Respondents were asked to rate on a 5-point scale from “1: Strongly disagree” to “5: Strongly agree” (Questionnaire).

5. Motivation to Learn

For motivation to learn, the Japanese version of the ARCS evaluation sheet was used, which is based on the ARCS motivational model proposed by Keller [19], which considers motivation to learn from four aspects [20,21]. The scale consists of 17 questions on factors that influence motivation to learn: Attention (4 items), Relevance (4 items), Confidence (4 items), and Satisfaction (5 items). The subjects were asked to rate their positive to negative impressions using the 5-point SD method (Questionnaire).

6. Evaluation of MR Materials

The subjects were asked to respond freely to questions about what they thought was good about the MR educational materials, what they would like to see improved, and what they would like to see requested.

### 2.5. Sample Size

Repeated measures analysis of variance was used to compare knowledge and comprehension before and after education in the Sim and MR groups. Using G*Power 3.1.9.7 with “F tests” and “ANOVA” statistics, an effect size of “0.25” (effect size: medium) and power of “0.8” from previous studies, the number of groups of “2” and the number of repetitions of “2”, a total of 34 cases, 17 in each group, were calculated.

### 2.6. Randomization

Participants who provided consent were randomly assigned to two groups using block randomization with a block size of four.

### 2.7. Statistical Method

The Mann–Whitney U test and χ^2^ test were used to confirm the variation in the background of the subjects in each group. Comparison of understanding, anxiety, and confidence regarding observation of postoperative patients before and after education and between groups was performed using two-way repeated measure ANOVA. The Mann–Whitney U test was used for time of educational intervention, satisfaction and self-confidence in learning, and motivation to learn. The number of observations conducted was compared using a two-sample *t*-test, and the number of clinical judgments conducted was compared using the Mann–Whitney U test. The χ^2^ test and Fisher’s direct test were used to compare the items observed in the evaluation of practices. Free descriptions were summarized so that the semantic content of the descriptions did not change and were categorized by checking for similarities and differences.

The statistical analysis software was IBM SPSS Statistics Version 29 (International Business Machines Corporation, New York, NY, USA), and the significance level of each was set to less than 5%.

### 2.8. Ethics

The purpose of the research and ethical considerations were explained using an explanatory document, and consent was obtained before the research was conducted. In addition, care was taken in the handling of personal information, including ensuring anonymity and protecting privacy. The subjects’ free decision to participate in this study and to withdraw their consent after consent was respected. In addition, the researchers were not in charge of evaluating the performance of all subjects, and they were asked to participate after explaining that their academic performance would not be disadvantaged. This study was approved by the Ethics Committee of the Hirosaki University Graduate School of Health Sciences (Reference number: 2024-004).

## 3. Results

### 3.1. Participants Flow

The 34 applicants were randomly assigned to two groups for the experiment. There were no exclusions.

### 3.2. Recruitment

The recruitment period for participation was from 10 June to 20 July 2024. An email describing the purpose of the study was sent to all eligible students, and subjects were recruited through their applications.

This study was conducted from 27 June to 2 August 2024. The experiment was terminated when the number of cases was reached.

### 3.3. Number Analyzed

A total of 34 applicants for this study were analyzed, with 17 classified into the Sim group and 17 into the MR group.

### 3.4. Outcome and Estimation

#### 3.4.1. Background of Subjects

The average age of the subjects was 21.4 ± 0.5 (mean ± SD). Regarding their practical experience in observing postoperative patients, 9 students (26.5%) had no experience, 19 students (55.9%) had seen the scene of observation, and 6 students (17.6%) had performed observations. Regarding their perception, 2 students (5.9%) answered that they were rather good at it, 20 students (58.8%) answered that they were rather poor at it, and 12 students (35.3%) answered that they were poor at it. There were no significant differences in the background of the subjects in the Sim and MR groups (Table 3).

#### 3.4.2. Contents and Effectiveness of Educational Interventions

1. Implementation Time

Comparing the educational time, the simulation time was 8.7 ± 8.0 min for the Sim group and 34.3 ± 9.6 min for the MR group, which was significantly longer in the MR group (*p* < 0.001). Debriefing time was significantly longer in the Sim group, 32.6 ± 10.3 min for the Sim group and 7.9 ± 3.9 min for the MR group (*p* < 0.001). There was no significant difference in total time (*p* = 0.786) (Table 4).

2. Comparison of Educational Effects by the Number of Observation and Postoperative Complication Judgment Items

Table 5 compares the number of observation and postoperative complication judgment items conducted by each group after the educational intervention. The number of observations performed was significantly higher in the MR group (43.5 ± 7.8 items in the Sim group and 52.5 ± 5.1 items in the MR group (*p* < 0.001). There was no significant difference in the number of postoperative complication items performed (*p* = 0.357) (Table 5).

3. Understanding, Anxiety, and Confidence

A comparison of pre- and post-education on understanding, anxiety, and confidence in observing postoperative patients indicated a significant increase in self-evaluation in all items for both groups. When compared between groups, the simulation group had significantly better ratings for “I can explain observation procedures”, “I am not anxious about the observation”, and “I am confident in my observations” (Table 6).

#### 3.4.3. Evaluation of Teaching Methods

1. Satisfaction and Self-Confidence in Learning

A comparison of the groups’ ratings of Satisfaction and Self-Confidence in Learning revealed the following results. The Sim group scored significantly higher on “The teaching methods used in this simulation were helpful and effective”. (*p* = 0.041), “The way my instructor(s) taught the simulation was suitable to the way I learn”. (*p* = 0.008), and “I am confident that I am mastering the content of the simulation activity that my instructors presented to me”. (*p* = 0.005). The other items had no significant differences (Table 7).

2. Motivation to Learn

**Figure 6:**
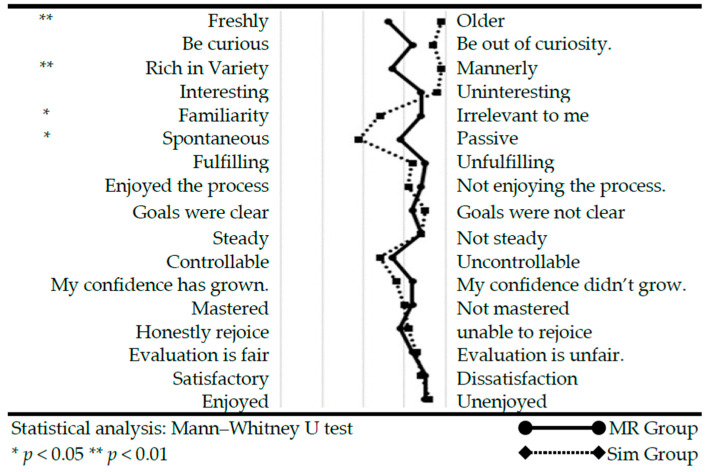
Comparison of motivation to learn.

A comparison of the groups’ ratings of their willingness to learn revealed the following results. The MR group was significantly more positive about “Fleshly–Older” (*p* < 0.001) and ”Rich in Variety–Mannerly” (*p* < 0.001). “Familiarity–Irrelevant to me” (*p* = 0.016) and “Spontaneous–Passive” (*p* = 0.031) were significantly positively evaluated by the Sim group. The other items had no significant differences (Figure 6).

#### 3.4.4. Evaluation of MR Materials

The results of categorizing the free responses about good points of the MR materials were classified into six categories: “Better than traditional teaching and learning materials”, “Learning effectiveness”, “Able to work on own initiative”, “Enjoyable to learn”, “Psychologically safe” and “Practical” (Table 8). In addition, free descriptions of “improvements and requests” for MR materials were categorized into four categories: “Difficult to use”, “Hard to see”, “Can’t get the whole picture”, and “Voluminous” (Table 9).

### 3.5. Harm

No harmful events in this experiment.

## 4. Discussion

### 4.1. Interpretation

In this study, a clinical judgment support MR program for postoperative patient observation was developed and compared to simulation education and educational effectiveness. As a result, more observation items were implemented in education using MR materials than in simulation education. In previous studies, nurses have systematically and efficiently observed patients based on their priorities [22]. In contrast, nursing students have little basic knowledge and practical experience in observing postoperative patients, and they have difficulty in practice, such as not knowing how to act and not having established a foundation for action [6]. In the MR teaching materials developed, experience with all the observation items led to establishing an image of observation, and practice with correct techniques while receiving hints enabled the acquisition of solid skills. Especially in education related to clinical judgment, think aloud, which conveys the thoughts of skilled nurses to learners, is effective [23]. In the MR materials designed to replicate the observational skills of professional nurses, it is believed that by practicing within the framework of the nurse’s systematic approach, learners can grasp the significance of the observation process and apply similar techniques in their practice. The aforementioned efficient learning materials are believed to have enabled students to observe more items. Furthermore, fidelity is important in simulation education, and reproducing real situations will better promote knowledge and skill development [24,25]. MR educational materials make it possible to create a projection of the actual patient’s condition on the simulator, thus adding a sense of reality. Given that clinical judgment skills improve through repeated experience [1], it is believed that experiential learning using real images in the MR teaching materials has facilitated learning that is directly applicable to clinical practice. For the debriefing, the times were shorter than for the simulation education. Debriefing is defined as “a place for learners to reflect on their actions, discuss areas for improvement, and incorporate new information into their previous knowledge” [26], and the process of making sense of the experience is developed [27]. Debriefing time is generally two to three times longer than the simulation [28]. The MR instructional material presents all procedures and provides hints during the simulation. As a result, the semantic process that usually occurs during debriefing can occur during simulation, thus reducing debriefing time. Free comments such as “The ability to reflect on the experience was very beneficial, allowing me to apply the learning to future situations” and the observed educational outcomes suggest that the material is highly effective. Research has shown that debriefing requires skilled personnel, burdens instructors significantly, and leads to variability in student learning outcomes based on the facilitator’s competence [4]. However, with MR educational materials, consistent learning outcomes are expected to be achieved regardless of the facilitator’s skill level while reducing personnel costs. As noted above, although the time allocated for simulation and debriefing was significantly different between MR materials and traditional simulation-based education, the time required was aligned with the unique characteristics of MR-based learning. Since there was no significant difference in the total time, MR was considered an efficient learning tool.

Considering the influence of the experimental method, it is conceivable that the MR group experienced all of the observation items in the educational intervention and thus may have been able to observe more items due to the holding of short-term memory. However, since there were 70 observation items for postoperative patients in this case study, and the structure of the items was complex, including consideration of priorities and practice of judgment, it would be difficult to conduct this study simply with short-term memory alone. Furthermore, some educational methods use demonstrations and video materials. However, the challenge lies in the fact that while images and understanding can be acquired, repeated practice is necessary to achieve mastery [29]. The MR materials may have had an educational effect because “It was easy to remember by doing it while actually doing it”. One student noted, “Even though I couldn’t memorize everything in one session, I was able to form a general idea of what it was like”. This was highlighted as a positive aspect, as students were able to actively engage in learning while building a mental image of the process. In addition, since the students practiced the items for which they did not know how to observe by looking at the hints and understanding the methods and meanings, they were able to practice with conviction from the beginning, which may have made it easier for them to remember and act on the information.

Regarding subjective effects, both groups improved their self-assessment of understanding, confidence, and anxiety regarding the observation of postoperative patients after education. However, confidence and anxiety improved significantly in the Sim group, and the MR group remained anxious after the education. According to previous studies, meta-cognition to objectively view one’s abilities is important because failure to recognize a lack of ability leads to the overestimation of one’s actions and situations [30]. The Sim group may be less aware of the items and skills required for postoperative observation than the MR group, which experienced all items. It is possible that the Sim group overestimated their competence because they were unaware of their lack of competence and developed a sense of confidence and satisfaction that they could observe better than before, even though their observations were inadequate. In the case of the MR group, on the other hand, giving them one observation item at a time and judging them led them to connect with their knowledge and become aware of their lack of knowledge and judgment. It is also thought that by first learning all the observation items and experiencing the practice, they were able to grasp the overall picture, which led to an objective grasp of their abilities. This may have helped them build a meta-cognition of their abilities to grasp the skills required to observe postoperative patients objectively. Therefore, it is possible that the subjects remained anxious because they objectively grasped that their observation was inadequate. However, as the nurses overcame this by becoming aware of their inexperience and growing [31], it is believed that this supported the students’ independent learning. However, the individualized debriefing in the simulation education may also have been an aspect that significantly increased satisfaction and confidence in learning. This has been shown in previous studies [32], but the program in the MR materials used in this study has limitations. Further educational benefits may be expected from improvements in MR materials, such as incorporating higher-order technologies such as artificial intelligence to enable individualized learner involvement.

Based on the above, the MR teaching materials enabled the students to learn the thinking of expert professionals through visual effects and to give them experience with actual images, resulting in improved skills in observing postoperative patients. Since learning the thinking of expert nurses and experiential learning are essential for clinical judgment [1], using MR technology in nursing training programs can provide learners with more practical experience. However, for techniques that require interactive interaction with the patient or when flexible judgment is needed in response to changing situations, the current program has limitations in setting up MR teaching materials, and conventional simulation education may be more effective.

Since simulation education causes anxiety and stress, it is important to ensure psychological safety [33,34]. It was stated, “It was easy to concentrate on the exercises because I did not have the oppressive feeling that someone (e.g., the teacher) was watching me closely”. Since the MR materials are materials that individual students can learn without having any psychological burden, on the other hand, the MR materials give the impression of passive learning. It is possible that the students in the Sim group felt spontaneous because they studied and practiced the observation items independently, while the students in the MR group felt passive because they followed the instructions. In addition, the students in the MR group mentioned the improvement point of “Cannot be able to grasp the whole picture”. It is believed that students in the MR group had a passive experience of merely following the observation process, as the core concepts and educational objectives related to postoperative patient observation—including observation items, procedures, and criteria for judgment—were not effectively communicated to them. However, the MR educational materials that we developed presented observation items but allowed the actual act of observation and judgment to be guided by the learner’s spontaneous actions, making the learning process inherently self-directed.

The students found the education using MR materials to be “Freshly” and “Rich in Variety”, with free descriptions such as “I enjoyed the novel, the near-future feel of it”, and “I felt that it lowered the bar for my studies”. The MR group encountered new equipment and a distinct learning experience, which provided a refreshing change for the students. Consequently, this exposure stimulated their interest, intellectual curiosity, and spirit of inquiry, concluding that the materials successfully captured the students’ attention. On the other hand, the program also revealed some points that need to be improved, such as the difficulty of operation and the lack of visibility.

### 4.2. Limitation

In this study, the MR group practiced all the observations immediately before the simulation. In contrast, the Sim group had one simulation and debriefing and did not necessarily practice all the observation items. It is possible that the MR group retained more short-term memory and was able to practice a more significant number of observation items. In addition, simulation education is based on experiential learning theory and is intended to conceptualize practice and consolidate knowledge and skills through cyclic learning by tackling similar tasks, so it is possible that a single practice session such as this may not be effective. In this regard, examining the effects of conducting education multiple times is necessary. In the future, it will be required to verify the educational long-term impact and the effects when education is repeated each time.

## 5. Conclusions

In this study, the effectiveness of the MR program to support clinical judgment in the observation of postoperative patients was examined by comparing it with conventional simulation education.

The MR program was an effective and efficient learning tool, allowing more practice through hands-on experiential learning and self-debriefing. In addition, metacognition was used for appropriate self-assessment and provided moderate confidence and satisfaction. Based on the above, the MR materials are expected to be more effective than simulation education in teaching clinical judgment regarding the observation of postoperative patients in some areas.

## Figures and Tables

**Table 1:** Case and task.

<Case> ○ Name: Hiroko Tanaka ○ Gender: Female ○ Age: 70 ○ Diagnosis: Gastric cancer ○ Medical History: Not applicable. ○ History of present illness: Since last year, she has been experiencing discomfort in the stomach area and has been taking over-the-counter stomach medicine. Since then, symptoms had not improved, and she had lost 10 kg of weight in 3 months, so she visited Hospital A. Gastric endoscopy, and biopsy revealed gastric cancer, and she was hospitalized for surgery. ○ Outline of Surgery [Surgical Method] Total Gastrectomy [Anesthesia Method] General Anesthesia [Time] 4 h [Blood Loss] 400 g After surgery, the patient was extubated in the recovery room, the arterial catheter was removed, and she was in good respiratory condition. Her general condition was stable, so that she returned to the hospital room an hour later. ○ Condition upon return to the hospital room Nasogastric tube, indwelling bladder catheter, and subhepatic drain being inserted (connected to drainage bag). A venous catheter was inserted in the left forearm, and the physician ordered IV fluids at a flow rate of 20 mL/h to nurses. Oxygen indication: 3 L/min (Oxygen mask) Activity Restriction Level: Complete bed rest <Task> As a nurse, observe the patient postoperatively and assess her general condition.

**Table 2:** 70 observation items and seven postoperative complications judgment items.

Observation Items	Observation Items
Awakening status	Concentration of drainage fluid
Vocalization	Smell of drainage fluid
Indicative action	Abdominal distention and fullness
Complexion	Rumbling stomach
Facial expression	Nausea/vomiting
Delirium	Fart
Oxygen inhalation method	Intestinal peristalsis
Oxygen flow rate	Redness of wound
Respiratory frequency	Swelling of wound
Pulmonary auscultation	Wound pain
Dyspnea	Fever of wound
Secretions in the airway	Bleeding from the wound
Sputum	Exudate from the wound
Chest pain	Level of pain (Numerical Rating Scale)
Respiratory depression	Location of pain
Root of the tongue sinking	Nature of pain
SpO2	Duration of pain
Fixed position of gastric tube	Redness of skin on the back
Fixed length of gastric tube	Redness of the skin in the sacral region
Skin in gastric tube fixation position	Redness of skin around the anus
Gastric tube drainage volume	Status of indwelling bladder catheter fixation
Color tone of gastric tube drainage	Volume of urination
Concentration of gastric tube drainage	Tone of urination
Types of ECG inductions	Peripheral pulse of the lower extremities
ECG Waveform	Pain in the lower extremities
ECG: Arrhythmia	Color tone of lower extremities
Pulse palpation	Cyanosis of the lower extremities
Tachycardia or bradycardia	Coldness in the lower extremities
Pulse palpation: Arrhythmia	Homann’s sign
Blood pressure	Erythema of the calcaneal region
High or low blood pressure	
Temperature	
Shivering	Postoperative Complication Judgment Items
Hypothermia (<35 °C)	Poor state of consciousness
Intravenous infusion instructions and status	Possible airway obstruction
Drop rate	Possible atelectasis
Condition of intravenous infusion puncture site	Possible postoperative bleeding
Type of drain	Problems due to postoperative pain
Drainage volume	Dermatologic integrative disorder
Color tone of drainage fluid	Abnormal blood data

**Table 3:** Background of subjects.

Item		Total	Sim Group	MR Group	*p*
Number		34	17	17	-
Average Age		21.41 ± 0.50	21.47 ± 0.51	21.35 ± 0.49	0.563
Practical Experience (%)	I had no experience.	9 (26.5)	4 (23.5)	5 (29.4)	0.660
	I had seen the scene of observation.	19 (55.9)	9 (53.0)	10 (58.8)	
	I had performed observations.	6 (17.6)	4 (23.5)	2 (11.8)	
Are you good or poor at observation? (%)	I’m good at it.	0 (0)	0 (0)	0 (0)	1.000
	I’m rather good at it.	2 (5.9)	1 (5.9)	1 (5.9)	
	I’m rather poor at it.	20 (58.8)	10 (58.8)	10 (58.8)	
	I’m poor at it.	12 (35.3)	6 (35.3)	6 (35.3)	

Statistical analysis: Mann–Whitney U test, Pearson’s chi-squared test.

**Table 4:** Implementation time.

Item	Sim Group	MR Group	*p*
Simulation time (min)	8.7 ± 8.0	34.3 ± 9.6	<0.001
Debriefing time (min)	32.6 ± 10.3	7.9 ± 3.9	<0.001
Total time (min)	41.3 ± 7.2	42.2 ± 11.6	0.786

Statistical analysis: Mann–Whitney U test.

**Table 5:** Number of observations and clinical decisions conducted.

Item	Sim Group	MR Group	*p*
Number of observations performed (items)	43.5 ± 7.8	52.5 ± 5.1	<0.001
Number of postoperative complication judgment items performed (items)	2.8 ± 1.8	3.5 ± 2.1	0.357

Statistical analysis: independent *t*-test, Mann–Whitney U test.

**Table 6:** Comparison of understanding, anxiety, and confidence in postoperative patient observation before and after education and between groups.

Item		Sim Group	MR Group	*p* **
I can explain observation procedures	pre	−1.2 ± 0.9	−1.2 ± 0.7	1.000
	post	0.9 ± 0.7	0.2 ± 0.8	0.005
	*p* *	<0.001	<0.001	
I can explain observation methods	pre	−1.0 ± 1.0	−1.1 ± 0.9	0.715
	post	0.9 ± 0.4	0.8 ± 0.7	0.570
	*p* *	<0.001	<0.001	
I can explain the judgment required to observe	pre	−1.0 ± 0.9	−1.0 ± 0.6	1.000
	post	0.9 ± 0.6	0.9 ± 0.6	0.769
	*p* *	<0.001	<0.001	
I am not anxious about the observation	pre	−1.7 ± 0.5	−1.4 ± 0.6	0.229
	post	0.0 ± 0.9	−0.7 ± 0.7	0.029
	*p* *	<0.001	0.002	
I am confident in my observations	pre	−1.7 ± 0.5	−1.4 ± 0.6	0.474
	post	0.0 ± 0.9	−0.7 ± 0.7	0.022
	*p* *	<0.001	<0.001	

Statistical analysis: two-way repeated measure ANOVA. *p* *, *p* **: Bonferroni.

**Table 7:** Comparison of satisfaction and self-confidence in learning.

Item	Sim Group	MR Group	*p*
The teaching methods used in this simulation were helpful and effective.	4.5 ± 0.5	4.1 ± 0.3	0.041
The simulation provided me with a variety of learning materials and activities to promote my learning the medical surgical curriculum.	4.4 ± 0.8	4.0 ± 0.5	0.079
I enjoyed how my instructor taught the simulation.	4.4 ± 0.5	4.0 ± 0.8	0.259
The teaching materials used in this simulation were motivating and helped me to learn.	4.2 ± 0.8	4.2 ± 0.7	0.760
The way my instructor(s) taught the simulation was suitable to the way I learn.	4.3 ± 0.7	3.5 ± 0.7	0.008
I am confident that I am mastering the content of the simulation activity that my instructors presented to me.	3.8 ± 0.8	2.7 ± 1.1	0.005
I am confident that this simulation covered critical content necessary for the mastery of medical surgical curriculum.	4.6 ± 0.5	4.5 ± 0.6	0.683
I am confident that I am developing the skills and obtaining the required knowledge from this simulation to perform necessary tasks in a clinical setting	4.5 ± 0.6	4.3 ± 0.5	0.339
My instructors used helpful resources to teach the simulation.	4.0 ± 0.8	4.1 ± 0.8	0.838
It is my responsibility as the student to learn what I need to know from this simulation activity.	4.1 ± 0.5	4.2 ± 0.7	0.563
I know how to get help when I do not understand the concepts covered in the simulation.	3.5 ± 0.9	3.4 ± 1.3	0.919
I know how to use simulation activities to learn critical aspects of these skills.	4.0 ± 0.7	3.7 ± 0.9	0.433
It is the instructor’s responsibility to tell me what I need to learn about the simulation activity content during class time.	4.5 ± 0.5	4.2 ± 1.0	0.658

Statistical analysis: Mann–Whitney U test.

**Table 8:** Free descriptions of good points about the MR materials.

Category (Code)	Code Example
Better than traditional teaching and learning materials (4)	In the past, I had to check a checklist on paper before moving on to the next action, but with the MR materials, I can check and move on to the next action without changing my perspective. It was good that I could see hints and images so I could work on my ideas until the end.
Learning effectiveness (3)	It was easy to remember by doing it while actually doing it. Even though I couldn’t memorize everything in one session, I was able to form a general idea of what it was like.
Able to work on own initiative (3)	I was able to proceed with the observation spontaneously. The ability to reflect on the experience was very beneficial, allowing me to apply the learning to future situations.
Enjoyable to learn (2)	I enjoyed the novel, the near-future feel of it I felt that it lowered the bar for my studies.
Psychologically safe (1)	It was easy to concentrate on the exercises because I did not have the oppressive feeling that someone (e.g., the teacher) was watching me closely.
Practical (1)	Unlike the exercises using mannequins, it was easier to visualize the clinical observations by seeing the images and making judgments.

**Table 9:** Free descriptions of improvements and requests for MR materials.

Category (Code)	Code Example
Difficult to use (4)	There were many cases of mistaken or incorrectly pressed buttons. I thought it would take time to get used to it.
Hard to see (3)	The display screen was not clear, depending on the QR code. The text was too thin and sometimes difficult to read.
Cannot be able to grasp the whole picture (2)	I wish the overall procedure was shown somewhere so that I would know where I am at and have an idea of the overall procedure. I was so focused on completing the problems that I didn’t have a clear impression of what order I was doing them in after I was done.
Voluminous (1)	I felt the amount of programs was too much.

## Data Availability

The original contribution presented in the study are included in the article/Supplementary Material, further inquiries can be directed to the corresponding author.

## Supplementary Materials

The following supporting information can be downloaded at: https://www.mdpi.com/article/10.3390/healthcare12232357/s1, Questionnaire: Questionnaire survey.
