# Supplementary material for: Development and Educational Effectiveness of a Mixed Reality (MR) Program to Support Clinical Judgment in the Observation of Postoperative Patients
Source: Healthcare (Basel). 2024 Nov 25;12(23):2357. doi: 10.3390/healthcare12232357 (PMC11641660; doi:10.3390/healthcare12232357)
Supplement: Supplementary file 1 [file healthcare-12-02357-s001.zip › healthcare-3286445-supplementary.pdf]

Questionnaire

No.______

1. Understanding, anxiety, and confidence regarding observation of postoperative patients

Please put a "○" in the box that applies.

No	Items	Strongly disagree	Disagree	Neither	Agree	Strongly agree
1	I can explain observation procedures.					
2	I can explain observation methods.					
3	I can explain the judgment required to observe.					
4	I am not anxious about the observation.					
5	I am confident in my observations.					

2. Satisfaction and Self-confidence in Learning

Please put a "○" in the box that applies.

No	Items	Strongly disagree	Disagree	Neither	Agree	Strongly agree
1	The teaching methods used in this simulation were helpful and effective.					
2	The simulation provided me with a variety of learning materials and activities to promote my learning the medical surgical curriculum.					
3	I enjoyed how my instructor taught the simulation.					
4	The teaching materials used in this simulation were motivating and helped me to learn.					
5	The way my instructor(s) taught the simulation was suitable to the way I learn.					
6	I am confident that I am mastering the content of the simulation activity that my instructors presented to me.					
7	I am confident that this simulation covered critical content necessary for the mastery of medical surgical curriculum.					
8	I am confident that I am developing the skills and obtaining the required knowledge from this simulation to perform necessary tasks in a clinical setting					
9	My instructors used helpful resources to teach the simulation.					
10	It is my responsibility as the student to learn what I need to know from this simulation activity.					
11	I know how to get help when I do not understand the concepts covered in the simulation.					
12	I know how to use simulation activities to learn critical aspects of these skills.					
13	It is the instructor's responsibility to tell me what I need to learn about the simulation activity content during class time.					

3. Motivation to Learn

Please put a "○" in the box that applies to an image of the education received

	Very	A little		A little	Very	
Freshly						Older
Be curious						Be out of curiosity.
Rich in Variety						Mannerly
Interesting						Uninteresting
Familiarity						Irrelevant to me
Spontaneous						Passive
Fulfilling						Unfulfilling
Enjoyed the process						Not enjoying the process.
Goals were clear						Goals were not clear
Steady						Not steady
Controllable						Uncontrollable
My confidence has grown.						My confidence didn't grow.
Mastered						Not mastered
Honestly rejoice						unable to rejoice
Evaluation is fair						Evaluation is unfair.
Satisfactory						Dissatisfaction
Enjoyed						Unenjoyed
